# Sexual Arousal in Men Who Have Sex Exclusively with Men: Its Relationship with Sexual Cognitions of Dominance and Submission

**DOI:** 10.3390/healthcare14121611

**Published:** 2026-06-08

**Authors:** Juan Carlos Sierra, Federico Mercedes-Jiménez, Carlos Pérez-Amorós, Laura Elvira Muñoz-García

**Affiliations:** Mind, Brain and Behavior Research Center (CIMCYC), University of Granada, 18011 Granada, Spain; federicomj02@correo.ugr.es (F.M.-J.); amoroscp@ugr.es (C.P.-A.); noselaura@correo.ugr.es (L.E.M.-G.)

**Keywords:** sexual cognitions, dominance, submission, sexual arousal, erection, men who have sex exclusively with men

## Abstract

**Background/Objectives**: The sexuality of LGBTQIA+ individuals has been less studied than that of heterosexuals. This study aimed to examine, in cisgender men who have sex exclusively with men, the association of positive (PSC) and negative (NSC) sexual cognitions of dominance and submission with sexual arousal and erection in the contexts of solitary masturbation and sexual relationships. **Methods**: A sample of 253 men, aged 18 to 67 years old, completed an online survey assessing the frequency of PSC and NSC and their sexual functioning in solitary masturbation and sexual relationships. Mixed linear models were used to analyze the association of PSC and NSC of dominance and submission with sexual arousal and erection in both contexts. **Results**: The results revealed, first, a lack of association between PSC and arousal/erection, except for a negative association with a submission PSC (“being sexually victimized”). Regarding NSC, on one hand, a positive relationship of a specific dominance cognition (“whipping or spanking someone”) with arousal and erection was found. On the other hand, a negative association of a specific submission cognition (“being whipped or spanked”) with arousal was found. Furthermore, the interaction of this NSC of dominance with the context was significant. **Conclusions**: The results obtained in this study highlight the complexity of the relationship between positive (PSC) and negative (NSC) sexual cognitions of dominance and submission with sexual arousal and erection in cisgender men who have sex exclusively with other men.

## 1. Introduction

Sexual fantasies are a source of sexual pleasure, defined as the physical and psychological enjoyment derived from shared or solitary erotic/sexual experiences, including sexual cognitions and fantasies [1]. They are described as mental representations and cognitions of an erotic or sexually stimulating nature for the person during the waking state [2]. Most people claim to have experienced sexual fantasies at some point in their lives [2,3,4]. Traditionally, sexual fantasies have been linked to a satisfying and active sex life and are associated with positive feelings due to their ability to cause sexual arousal [5,6]. However, this is not always the case, as they can also be accompanied by feelings of guilt and discomfort, even if they cause sexual arousal [7]. Renaud and Byers [8] use the term “sexual cognition” to distinguish between these two possible experiences of sexual fantasies (i.e., accompanied by positive or negative affect). In this sense, these authors differentiate between positive sexual cognitions (PSC), i.e., experienced as acceptable, pleasant, and ego-syntonic, and negative sexual cognitions (NSC), considered as unacceptable, unpleasant, and ego-dystonic. The impact of both types of cognitions on sexual health is different, with PSC generally being associated with higher sexual functioning, whereas NSC show negative or non-significant associations [9,10].

Traditionally, sexual fantasies have been classified, based on their content, as intimate, exploratory, sadomasochistic, and impersonal [11]. Intimate sexual fantasies are associated with a deep relationship and emotional connection with a significant other with whom one has an emotional bond; exploratory fantasies are related to the search for new and varied sexual experiences; sadomasochistic fantasies focus on power dynamics, encompassing dominant and submissive sexual behaviors, which may involve pain; and, finally, impersonal fantasies refer to sexual manifestations that give little importance to feelings due to their lack of emotional involvement [11]. Moyano and Sierra [12] used this classification to categorize PSC and NSC assessed by the Sexual Cognitions Checklist (SCC) in such a way that sexual cognitions associated with positive or negative affections can be intimate, impersonal, sadomasochistic, or exploratory. Although the original authors did not originally examine the factorial structure of the SCC, they identified six dominance items (e.g., “tying someone up”) and ten submission items (e.g., “being tied up”) that showed acceptable internal consistency coefficients [13,14]. In line with this distinction, sadomasochistic cognitions can be further differentiated into sexual cognitions of dominance and submission, depending on the role adopted [3,13,14,15,16,17]. Dominance-related cognitions involve the erotic mental representation of exerting control over another person, whereas sexual submission thematics involve being forced or sexually subdued, relinquishing control, or adopting a passive or vulnerable role [2].

Several studies have adopted this subdivision and examined its associations with sociodemographic factors, sexual functioning, and, particularly, experiences of sexual aggression or victimization (e.g., [3,13,14,15,16,17,18]) observing, for example, that people who report a history of sexual coercion have a higher frequency of dominance PSC [13,15], while those who have been victims of sexual abuse in childhood or adulthood report a higher frequency of submission PSC [14,15]. However, most of this research has been conducted in cisgender heterosexual samples, with limited attention to sexually diverse populations.

In addition, research outside the SCC framework highlights the relevance of dominance- and submission-related content in general-population fantasies. Evidence points to a high prevalence of BDSM (bondage, discipline, dominance, submission, sadism, and masochism)-related fantasies and interests despite substantially lower rates of enactment [19,20,21], a pattern also observed in Spanish samples [4]. These contents involve a strong interpersonal component based on negotiated power dynamics and have been linked to attachment styles [22], while also being frequently experienced at the level of fantasy, making them particularly suitable for examining both solitary and partnered sexual contexts. Furthermore, although traditionally approached from pathologizing perspectives [20,21], contemporary research demonstrates that they are relatively common [4,19,21,23] and not necessarily associated with maladaptive functioning, challenging their historical characterization as “deviant”. As Joyal et al. [21] suggest, the focus should be on the effect of a sexual fantasy rather than its content. In this sense, distinguishing between PSC and NSC allows for a more nuanced approach, acknowledging that dominance and submission content may be experienced as either pleasurable and ego-syntonic or distressing and ego-dystonic.

While sexual fantasies and cognitions have the capacity to elicit sexual arousal [24,25], few studies have linked PSC and NSC to this construct, and findings remain inconsistent, largely due to differing conceptualizations of sexual arousal. The results of these studies indicate, on the one hand, a positive relationship between sadomasochistic PSC and the propensity for sexual arousal [9]; while sadomasochistic NSC are associated with lower sexual arousal in the contexts of solitary masturbation and sexual relationships [10]. More recent findings observed that dominance-related PSC are positively associated with sexual arousal, whereas both submission and dominance NSC show negative associations [18]. However, none of these studies have included sexually diverse populations. As these types of sexual cognitions involve power-related and interpersonal dynamics, they may be particularly well suited for examining sexual arousal across both solitary and partnered contexts.

The sexuality of LGBTQIA+ (Lesbian, Gay, Bisexual, Transgender, Queer/Questioning, Intersex, and Asexual) individuals has historically been less studied than that of heterosexual populations, often remaining in the background of research [26]. Nevertheless, differences based on sexual orientation have been documented across multiple dimensions of sexuality, including sexual attitudes [27], subjective orgasmic experience [28], and sexual functioning [29]. Evidence also suggests variability in the frequency and content of sexual fantasies and cognitions as a function of sexual orientation, although this evidence has been primarily examined without distinguishing between PSC and NSC, particularly within dominance- and submission-related cognitions. Within sexual orientation, Sierra et al. [17] reported descriptive trends indicating that gay men tend to show higher scores across most PSC and NSC of dominance- and submission-related cognitions compared to lesbian women, with submission NSC representing the only exception. Although these differences were not systematically tested across all subtypes and should therefore be interpreted cautiously, they provide preliminary evidence suggesting that these cognitions may be more frequently endorsed in gay men.

In line with these findings, gay men tend to report greater openness and diversity in sexual fantasies, particularly those related to power roles such as domination or submission [30]. Specifically, compared to heterosexual men, gay men report more fantasies involving submissive behaviors (e.g., being strangled by their partner during sex) and fewer involving active domination (e.g., physically assaulting or controlling their partner) [17,31]. However, other studies have shown that gay men not only report a higher frequency of dominance and submission fantasies [32] but also exhibit a more versatile repertoire, integrating both active and passive roles within their sexual imagery [33]. In a similar vein, Brown et al. [34] reported that non-heterosexual participants tend to show greater arousal to fantasies involving rough sex, submission/masochism, and degradation/humiliation. Importantly, Canivet et al. [35] observed that gay participants were more likely to report domination and violent perpetration fantasies associated with high shame. Overall, the literature regarding dominance- and submission-related sexual cognitions in gay men remains largely focused on sexual fantasies without distinguishing between PSC and NSC, and findings have shown inconsistent patterns across studies. These inconsistencies highlight the need for further research to clarify how these cognitions are expressed and how they relate to sexual arousal in this population.

Sexual orientation is conceptualized as a multidimensional and potentially fluid construct, with self-reported current sexual behavior representing one of its core components [36,37]. Considering that sexual orientation and sexual behavior do not always fully overlap, as individuals may identify with a given orientation while reporting different patterns of sexual behavior [38], the present study adopts a behavioral operationalization of sexual orientation to reduce heterogeneity in sample definition. Therefore, to ensure conceptual clarity, the present study focuses on cisgender men who have sex exclusively with men. While terms such as “gay men” are used when referring to prior literature, participants are defined based on self-reported current sexual behavior. Additionally, the study focuses on cisgender participants given that the measures employed include physiological aspects of sexual response (e.g., sexual arousal and erection), which require consistency in anatomical assumptions to ensure valid interpretation. This aims to maintain comparability in sexual response measures and to avoid extending interpretations to populations with potentially different anatomical and physiological contexts, such as transgender and gender-diverse populations.

Therefore, the objective of this study is to examine, in men who have sex exclusively with men, the association of PSC and NSC of dominance and submission with self-reported sexual arousal, both globally (arousal: ease of becoming sexually aroused) and specifically (erection: ability to achieve and maintain an erection). Given the evidence that patterns of sexual arousal may differ between the contexts of solitary masturbation and sexual relationships (see Adell-Peña et al. [39]), both contexts are considered independently in this study. In this regard, we hypothesize that, in both the context of solitary masturbation and sexual relationships, we will find (a) a positive association of dominance and submission PSC with sexual arousal and erection, and (b) an absence of relationship, or a negative relationship, between dominance and submission NSC and sexual arousal and erection [9,10,18]. Dominance and submission dimensions were examined separately within both PSC and NSC. In addition to the hypotheses formulated—and due to the lack of previous evidence—the following research question is proposed: Will the interaction between PSC and NSC × context be significantly associated with sexual arousal and erection?

## 2. Materials and Methods

### 2.1. Participants

The inclusion criteria were being a cisgender man, being 18 years of age or older, being of Spanish nationality, and having, in the past 12 months, masturbated alone and engaged exclusively in sexual relations with other men. An a priori sample size estimate was conducted using simulation-based power analysis with the simr package (version 1.0.7) [40]. This approach was selected because the analysis used linear mixed-effects models with repeated-measures data nested within participants. The simulations were based on the planned model structure, including context, dominance, and submission dimensions, their interactions with context, and a random intercept for the participant. Assuming a medium effect size (β = 0.30) and α = 0.05, the results indicated that approximately 150 participants would be required to achieve 80% power. The final sample size (*N* = 253) exceeded this requirement.

Of the total participants who provided informed consent (*N* = 1289), the initial sample included individuals who did not meet the study’s target population (e.g., women and participants not fulfilling the inclusion criteria). After applying these criteria, 530 cisgender men were retained. From these, 510 provided complete sociodemographic data, 346 completed at least 75% of the responses for the sadomasochistic PSC and NSC measures, 296 completed the ASEX arousal and erection items in the solitary masturbation context, and, lastly, 253 completed the ASEX arousal and erection items in the sexual relationships context.

### 2.2. Measures

Sociodemographic and Sexual History Questionnaire. Collects information on nationality, sex, gender, age, educational level, type of sexual relationships (with people of different and/or the same sex), age of first sexual relationship, relationship status, and duration of relationship.

Subscale of sadomasochistic sexual cognitions from the Spanish version of the Sexual Cognitions Checklist (SCC) [12]. Consisting of seven items, three of which relate to sexual behaviors of dominance (e.g., “Forcing someone to do something sexually”) and four to submission (e.g., “Being pressured into engaging in sex”). They are rated as acceptable/pleasant (positive) and unacceptable/unpleasant (negative) on a 7-point Likert scale from 0 (*I have never had this cognition*) to 6 (*I have had (have) this cognition frequently during the day*), thus obtaining two scores: sadomasochistic PSC and NSC. The internal consistency reliability reported by Moyano and Sierra [12] was 0.81 for PSC and 0.85 for NSC. The factorial structure of the SCC has recently been validated in a sexually diverse population by Pérez-Amorós et al. [41]. In the present study, Cronbach’s alpha coefficients of 0.71 for PSC and 0.90 for NSC were obtained.

Spanish version of the Arizona Sexual Experience Scale (ASEX) [42] by Sánchez-Fuentes et al. [43]. Its five items assess subjective self-reported sexual functioning associated with sexual desire, sexual arousal, penile erection, vaginal lubrication, ability to reach orgasm, and satisfaction with orgasm during the last year. In gay men, its reliability was 0.77 [29]. In this study, the scale was answered separately for the context of solitary masturbation and for sexual relationships, and only items related to sexual arousal (“How easily are you aroused?”) and erection (i.e., “Can you easily get and keep an erection?”) were considered. Although the ASEX was designed as a multidimensional scale, for the purposes of this study, we specifically selected the items corresponding to sexual arousal and penile erection. This approach is justified by our focus on the physiological response associated with sexual cognitions. In the Spanish validation, these items have shown high item–total correlations (*r* = 0.81 and 0.79, respectively) and robust factor loadings (λ = 0.77 and 0.73, respectively) in exploratory factor analyses [43], suggesting that they capture a significant proportion of the variance of their respective constructs even when analyzed independently.

### 2.3. Procedure

The survey, hosted online on the Limesurvey platform (version 6.5.18+240723, Limesurvey GmbH, Hamburg, Germany) and located on the servers of the University of Granada, was distributed via social media, websites, mailing lists, and dissemination materials (e.g., posters and flyers) between March 2024 and April 2025. Recruitment targeted both the general population and the LGBTQIA+ community. Within the LGBTQIA+ community, recruitment was further supported through collaboration with LGBTQIA+ associations, which disseminated the survey through their mailing lists and social media channels. To control access to the survey and filter out possible automated responses, participants completed a CAPTCHA consisting of a simple random arithmetic operation. Participants were presented with an informed consent form detailing the study’s purpose, procedures, and data protection measures. Participation was voluntary and anonymous. No identifying personal data was collected, and only demographic information was recorded. Data confidentiality was ensured by stating that data would be used exclusively for scientific purposes, would not be shared with third parties, and would be accessible only to the research team. Data were stored securely on the University of Granada servers with restricted access. Once consent was given and the survey was completed, the responses were carefully reviewed to rule out any cases with inconclusive responses or abnormal patterns. A control item was used to ensure response validity. The study was approved by the Ethics Committee on Human Research at the University of Granada (Reference No. 3150/CEIH/2023).

### 2.4. Data Analysis

First, missing values in both SCC measures (i.e., sadomasochistic PSC and NSC) represent 0.90% of the data and were imputed using the missForest package (version 1.5) [44], through a decision tree-based algorithm that creates a random forest model for each variable based on observed values. Next, the normality of the study variables was assessed using the Shapiro–Wilk test, revealing significant deviations (*p* < 0.001). However, given the sample size and the sensitivity of this test, these results were interpreted with caution. As the main analyses relied on linear mixed-effects models, the normality assumption was evaluated at the level of model residuals. Residual diagnostics indicated an approximately normal distribution for both models (arousal: *W* = 0.994, *p* = 0.037; erection: *W* = 0.976, *p* < 0.001), supported by visual inspection of Q–Q plots. Additional assumptions, including linearity, homoscedasticity, and absence of multicollinearity or influential observations, were also met. Subsequently, the explanatory power of the PSC and NSC of dominance and submission was examined using mixed linear models (MLMs) for each repeated variable of sexual functioning (arousal and erection) in the contexts of solitary masturbation and sexual relationships. To do this, two mixed linear models (MLMs) were fitted. In the first, the dependent variable was the arousal score in the contexts of solitary masturbation and sexual relationships; in the second, the dependent variable was the erection score in the aforementioned contexts. In both models, the independent variables included the PSC and NSC of dominance and submission. Age and relationship status were initially examined as potential covariates in the mixed linear models; however, they were not statistically significant and did not alter the pattern of results and were therefore excluded from the final models to ensure parsimony. Finally, to determine the explanatory power of specific cognitions on arousal and erection variables, two additional MLMs were applied in which dominance and arousal items were considered as independent variables, while maintaining the dependent variables of arousal and erection in both contexts. To perform these analyses, the lme4 (version 1.1-37) [45] and lmerTest (version 3.2-0) [46] packages were used with the RStudio interface (version 2022.07.2+576, Rstudio PBC, Boston, MA, USA [47]).

## 3. Results

### 3.1. Sociodemographic Characteristics of Participants

The sociodemographic characteristics of the study participants are summarized in Table 1.

### 3.2. Association of Dominance and Submission PSC and NSC with Sexual Arousal

Only one significant negative association was found between sexual arousal and the total frequency of submission PSC (β = −0.116; *p* = 0.046). No significant associations were identified between interactions between sexual cognitions and context and sexual arousal. See Table 2.

Regarding specific sexual cognitions, sexual arousal was only associated with the submissive PSC “being sexually victimized” (β = −0.200, *p* = 0.032), the dominance NSC “whipping or spanking someone” (β = 0.236, *p* = 0.002), and the submission NSC “being whipped or spanked” (β = −0.164, *p* = 0.015). None of the interactions between specific sexual cognitions and context showed significant associations with sexual arousal. See Table 3.

### 3.3. Association of Dominance and Submission PSC and NSC with Erection

Regarding the association of the frequency of dominance and submission PSC and NSC with erection, no significant relationship was found between them or their interaction with context and erection, as can be seen in Table 4.

However, regarding the association between the frequency of each dominance and submission PSC and NSC, erection was only significantly positively associated with the dominance NSC “whipping or spanking someone” (β = 0.180, *p* = 0.032) as well as its interaction with context (β = 0.160, *p* = 0.038), as can be seen in Table 5.

**Figure 1:**
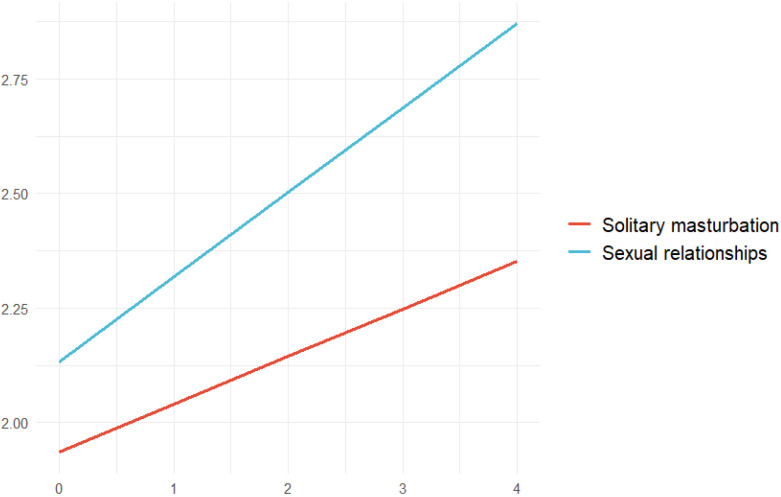
Association of interaction between NSC “whipping or spanking someone” and sexual context with erection. The *x*-axis represents the frequency of thoughts about “whipping or spanking someone”, while the *y*-axis represents the predicted levels of erection derived from the mixed-effects model.

The significant positive interaction observed between the NSC “whipping or spanking someone” and context suggests that the association between the frequency of negative cognition and erection varies depending on the context. Subsequent simple effects analyses indicated that the association between the NSC “whipping or spanking someone” and erection differed depending on the context, being stronger in the relational context than in the masturbation context, as illustrated in Figure 1.

## 4. Discussion

This study aimed to examine the association between dominance and submission PSC and NSC with sexual arousal and erection in the contexts of solitary masturbation and sexual relationships in cisgender men who have sex exclusively with other men. However, there are no studies that have specifically addressed these cognitions in this population. Based on previous studies conducted in heterosexual people [9,10,18], it was hypothesized that while PSC would be positively associated with sexual arousal and erection, NSC would either not be associated or would be negatively associated.

The results obtained in this study, conducted on men who have sex exclusively with men, do not support the first hypothesis, as dominance and submission PSC seem to be largely unrelated to sexual arousal or erection. Notably, when associations were observed (total frequency of submission PSC and the specific cognition of “being sexually victimized”), they were negative in direction. Importantly, the present findings contrast with prior evidence in heterosexual samples showing a positive association between submission PSC and sexual arousal and erection [18], suggesting that submission can be erotically functional rather than inhibitory. Consequently, the present findings may reflect sample-specific mechanisms rather than a general lack of erotic charge of submissive cognitions in men who have sex exclusively with men.

The negative association observed aligns with the framework proposed by Bivona and Critelli [48], which conceptualizes sexual cognitions related to rape as situated on an erotic–aversive continuum. Within this framework, sexual fantasies are neither conceptualized as purely pleasurable nor purely unpleasant but can mix pleasure with negative emotions. Thus, according to these authors, one could experience aversive erotic rape fantasies (characterized by genuine rejection, high levels of verbal or physical resistance, and negative emotions), erotic rape fantasies (characterized by feigned consent, low levels of verbal or physical resistance, and positive emotions), and erotic–aversive rape fantasies (characterized by genuine rejection, low levels of verbal or physical resistance, and both positive and negative emotions). On this continuum, it is possible that the PSC “being sexually victimized” may be positioned closer to the erotic–aversive category, such that certain aversive cognitive content, in this case manifesting as positively experienced sexual cognitions, may be associated with lower sexual arousal [49]. The fact that the negative association is observed in sexual arousal but not in erection suggests that the concordance between subjective sexual arousal and erection is low, coinciding with previous works [50]. Another tentative explanation may involve sexual role-related processes. Men who have sex exclusively with men may experience tensions derived from incongruence between their preferred sexual role and the role enacted or expected within specific encounters [51]. When present, such role-related tensions may generate distressing experiences that, in the domain of sexual cognitions, could interfere with the positive affective value of submissive sexual cognitions. Further research is needed to clarify these and other potential mechanisms, thereby enhancing our understanding of these findings. Therefore, these results should not be interpreted as evidence that submission PSC lack erotic value in men who have sex exclusively with other men.

Regarding NSC, it was hypothesized that there would be no relationship, or a negative relationship, between these cognitions and arousal/erection. The results largely support this hypothesis, except for the NSC “whipping or spanking someone”, which was found to be positively associated with both arousal and erection. As hypothesized, the overall NSC scores for dominance and submission did not correlate with arousal and erection, and the NSC “being whipped or spanked” was negatively associated with erection, results that are in line with those reported by Moyano et al. [9] and Pérez-Amorós et al. [10] in heterosexual populations. These findings, showing a pattern of null, positive, and negative associations rather than exclusively negative associations as initially hypothesized, may be interpreted within the aforementioned framework of an erotic–aversive continuum [48], as discussed above, possibly suggesting that BDSM-related practices do not show a uniform association with sexual response. The clearest example is seen in the NSC related to whipping or being whipped. Although both cognitions refer to similar practices, the discrepancy in the results may be related to the role assumed in the fantasy (active vs. passive). Thus, while the active cognition (“whipping or spanking someone”) may be experienced as erotic, the passive cognition (“being whipped or spanked”) could be associated with negative emotional responses, including anxiety or the reactivation of past traumatic experiences.

It is worth noting the theme around which the association of NSC with arousal and erection in cisgender men who have sex with men revolves: the anal region. Scientific literature has highlighted how the anal region plays a central role in gay men’s sexuality, being the object of a wide variety of sexual practices beyond penetration, such as rimming, fingering, or the use of insertive sex toys [52]. The relationship of these NSC with sexual arousal could be associated with the high erotic charge of this area of the body.

Finally, concerning the research question posed about whether the interaction between sexual cognitions of dominance/submission and context would be associated with arousal/erection, the answer provided by the results is that there is no association, except for the specific NSC “whipping or spanking someone,” which is related to a higher level of erection in the context of sexual relationships than in masturbation. This finding suggests that certain sexual cognitions may have a greater erotic charge when placed in an interpersonal context, which includes factors such as bodily feedback, mutual validation, and the activation of shared sexual scripts [53]. In this sense, beyond the need for another person to carry out the fantasy, despite the sexual cognition being perceived as negative, the physiological response could be intensified by the symbolic and emotional interaction with the sexual partner, who not only participates in the act but also may legitimize the content of the cognition, potentially reinforcing its arousal potential.

The results obtained in this study should be interpreted in light of certain methodological limitations. First, it is worth noting the sample size and the procedure chosen to select participants, which did not follow a random sampling of the population but rather a recruitment method involving the publication of an online survey via social media, websites, and mailing lists. Although the sample size was adequate for the main analyses, simulation-based power analysis suggested moderate power to detect the observed NSC × context interaction (70%, 95% *CI* [60.02, 78.76]). Therefore, non-significant interaction effects should be interpreted cautiously, as the study may have had limited power to detect smaller moderation effects. Most interaction effects between sexual cognitions and context were not statistically significant. While our study was powered to detect medium effects, we cannot rule out the existence of very small interaction effects that may have gone undetected due to limited statistical power. Detecting subtle interactions in human sexuality often requires larger, more diverse samples. Therefore, our null findings regarding interactions should be interpreted as an absence of moderate-to-large moderating effects of context, but future research with larger cohorts might be needed to confirm these results for more nuanced associations. Although linear mixed-effects models appropriately account for the hierarchical structure of the data and the non-independence of observations, the inclusion of multiple item-level predictors and interaction terms increases the number of statistical tests conducted. This may elevate the risk of Type I error, particularly in the item-level analyses, and therefore these findings should be interpreted with caution.

Some limitations regarding sampling are that most participants had a high level of education, and the assessment of sexual orientation used a behavioral criterion within a 12-month window rather than a multidimensional approach. While this increased sample homogeneity, it may have limited the capture of the full complexity of sexual orientation. Therefore, future research should incorporate more heterogeneous samples and multidimensional assessments of sexual orientation, as well as measures of sexual aggression/victimization, preferred sexual role, and substance use in sexual contexts alongside PSC and NSC. This would allow for a more comprehensive understanding of how diverse factors may influence the relationship between sexual cognitions and sexual arousal. Another methodological limitation involves the use of isolated items from the ASEX to measure sexual arousal and erection, rather than the full multidimensional scale. Although these items demonstrated strong psychometric indicators, including high factor loadings and robust correlations with the total scale score in previous validations, their use outside the complete instrument’s structure may limit the assessment of internal consistency for these specific dimensions. This choice was justified by our focus on specific physiological and subjective components of the sexual response; however, future studies should consider employing the full validated scale or incorporating objective measures (e.g., penile plethysmography) to complement these self-reported data and provide a more comprehensive view of sexual functioning.

## 5. Conclusions

The results obtained in this study highlight the complexity of the relationship between positive (PSC) and negative (NSC) sexual cognitions of dominance and submission with sexual arousal and erection in Spanish cisgender men who have sex exclusively with other men. Contrary to the hypothesis, PSC are not positively associated with arousal and erection; moreover, the submissive PSC “being sexually victimized” is negatively associated with sexual arousal. On the other hand, NSC show a more complex association with sexual arousal (i.e., no relationship, negative relationship, and positive relationship). While dominance-related cognition (“whipping or spanking someone”) is positively associated with arousal and erection, the cognition of submission (“being whipped or spanked”) is negatively associated with erection. These results underscore the potential importance of the role assumed in sexual relationships and its possible association with physiological response, given that contexts of submission can trigger negative emotions. Along these lines, the centrality of content linked to the anal region reveals a high erotic charge attributed to this body area within the sexuality of men who have sex exclusively with men. Finally, the positive association of certain negative sexual cognitions (e.g., “whipping or spanking someone”) with sexual arousal could be intensified in interpersonal contexts, where interaction, mutual validation, and shared sexual scripts may accentuate their excitatory potential.

Taken together, these findings highlight the need to develop clinical and preventive interventions that promote discrimination between healthy and free sexual practices and those that threaten people’s integrity. Similarly, the importance of depathologizing erotic diversity is emphasized.

## Figures and Tables

**Table 1:** Sociodemographic characteristics of participants (*N* = 253).

Sociodemographic Characteristics	
Age range and *M* (*SD*)	18–67, 32.27 (10.34)
Education level *n* (%)	
Technical training	47 (18.6)
Secondary education	6 (2.4)
University	200 (79.0)
Age of first sexual relationship *M* (*SD*)	17.72 (4.16)
Currently in a relationship *n* (%)	
Yes	136 (53.8)
No	117 (46.2)
Relationship duration in months *M* (*SD*)	77.63 (78.71)

Note. *M* = mean, *SD* = standard deviation.

**Table 2:** Association between total dominance and submission PSC and NSC frequency and sexual arousal.

Variable	Estimate	Standard Error	95% *CI*	*t*	*p*
(Intercept)	2.458	0.098	[2.26, 2.65]	24.992	<0.001
Dominance PSC	−0.049	0.065	[−0.01, 0.08]	−0.758	0.449
Submission PSC	−0.116	0.059	[−0.23, 0.00]	−2.003	0.046
Dominance NSC	0.004	0.081	[−0.15, 0.16]	0.048	0.962
Submission NSC	−0.045	0.074	[−0.10, 0.19]	0.608	0.544
Context	0.104	0.105	[−0.10, 0.31]	0.987	0.324
Dominance PSC × context	−0.015	0.069	[−0.15, 0.12]	−0.210	0.834
Submission PSC × context	−0.011	0.062	[−0.13, 0.11]	−0.171	0.865
Dominance NSC × context	−0.015	0.086	[−0.19, 0.15]	−0.177	0.860
Submission NSC × context	−0.004	0.079	[−0.16, 0.15]	−0.047	0.962

Note. PSC = positive sexual cognition; NSC = negative sexual cognition; *CI* = confidence interval.

**Table 3:** Association between the frequency of each dominance and submission PSC and NSC and sexual arousal.

Variable	Estimate	Standard Error	95% *CI*	*t*	*p*
(Intercept)	2.476	0.109	[2.26, 2.69]	22.768	<0.001
Context	−0.059	0.054	[−0.16, 0.05]	−1.103	0.270
Dominance PSC					
Forcing someone to do something sexually	0.024	0.053	[−0.08, 0.13]	0.445	0.657
Whipping or spanking someone	−0.074	0.043	[−0.16, 0.01]	−1.730	0.084
Forcing another adult to engage in a sexual act with me	−0.034	0.078	[−0.08, 0.22]	0.898	0.369
Forcing someone to do something sexually × context	−0.034	0.058	[−0.15, 0.08]	−0.581	0.561
Whipping or spanking someone × context	−0.009	0.047	[−0.10, 0.08]	−0.209	0.834
Forcing another adult to engage in a sexual act with me × context	0.110	0.086	[−0.06, 0.28]	1.283	0.200
Submission PSC					
Being pressured into engaging in sex	0.061	0.060	[−0.06, 0.18]	1.03	0.303
Being forced to do something sexually	−0.059	0.064	[−0.18, 0.07]	−0.925	0.355
Being sexually victimized	−0.200	0.094	[−0.38, −0.02]	−2.145	0.032
Being whipped or spanked	−0.025	0.043	[−0.11, 0.06]	−0.569	0.569
Being pressured into engaging in sex × context	0.057	0.066	[−0.07, 0.19]	0.874	0.383
Being forced to do something sexually × context	−0.036	0.069	[−0.17, 0.10]	−0.521	0.602
Being sexually victimized × context	−0.035	0.103	[−0.24, 0.17]	−0.340	0.734
Being whipped or spanked × context	0.021	0.048	[−0.07, 0.12]	0.444	0.657
Dominance NSC					
Forcing someone to do something sexually	−0.039	0.054	[−0.14, 0.07]	−0.719	0.472
Whipping or spanking someone	0.236	0.075	[0.09, 0.38]	3.127	0.002
Forcing another adult to engage in a sexual act with me	−0.034	0.056	[−0.14, 0.08]	−0.606	0.545
Forcing someone to do something sexually × context	−0.096	0.059	[−0.21, 0.02]	−1.617	0.107
Whipping or spanking someone × context	0.128	0.083	[−0.04, 0.29]	1.540	0.124
Forcing another adult to engage in a sexual act with me × context	0.058	0.061	[−0.06, 0.28]	0.943	0.346
Submission NSC					
Being pressured into engaging in sex	0.025	0.059	[−0.09, 0.14]	0.427	0.669
Being forced to do something sexually	0.096	0.064	[−0.03, 0.13]	1.507	0.132
Being sexually victimized	0.022	0.055	[−0.09, 0.13]	0.401	0.689
Being whipped or spanked	−0.164	0.067	[−0.30, −0.03]	−2.443	0.015
Being pressured into engaging in sex × context	−0.051	0.065	[−0.18, 0.08]	−0.782	0.435
Being forced to do something sexually × context	0.102	0.070	[−0.04, 0.24]	1.447	0.149
Being sexually victimized × context	−0.030	0.060	[−0.15, 0.09]	−0.501	0.617
Being whipped or spanked × context	0.005	0.074	[−0.14, 0.15]	0.065	0.948

Note. PSC = positive sexual cognition; NSC = negative sexual cognition; *CI* = confidence interval.

**Table 4:** Association between total dominance and submission PSC and NSC frequency and erection.

Variable	Estimate	Standard Error	95% *CI*	*t*	*p*
(Intercept)	1.998	0.107	[1.79, 2.21]	18.816	<0.001
Dominance PSC	0.082	0.070	[−0.05, 0.22]	1.180	0.238
Submission PSC	−0.051	0.063	[−0.17, 0.07]	−0.809	0.419
Dominance NSC	0.044	0.087	[−0.13, 0.21]	0.500	0.617
Submission NSC	0.011	0.080	[−0.15, 0.17]	0.132	0.895
Context	−0.234	0.098	[−0.43, −0.04]	−2.393	0.017
Dominance PSC × context	0.054	0.064	[−0.07, 0.18]	0.835	0.404
Submission PSC × context	−0.064	0.058	[−0.18, 0.05]	−1.104	0.270
Dominance NSC × context	−0.109	0.084	[−0.27, 0.05]	−1.364	0.173
Submission NSC × context	0.088	0.074	[−0.06, 0.23]	1.190	0.235

Note. PSC = positive sexual cognition; NSC = negative sexual cognition; *CI* = confidence interval.

**Table 5:** Association between the frequency of each dominance and submission PSC and NSC and erection.

Variable	Estimate	Standard Error	95% *CI*	*t*	*p*
(Intercept)	1.896	0.120	[1.66, 2.13]	15.811	<0.001
Context	0.229	0.050	[0.13, 0.33]	4.566	<0.001
Dominance PSC					
Forcing someone to do something sexually	0.061	0.059	[−0.05, 0.18]	1.043	0.297
Whipping or spanking someone	−0.001	0.048	[−0.09, 0.09]	−0.020	0.984
Forcing another adult to engage in a sexual act with me	0.075	0.087	[−0.10, 0.25]	0.859	0.391
Forcing someone to do something sexually × context	−0.005	0.054	[−0.11, 0.10]	−0.092	0.927
Whipping or spanking someone × context	−0.030	0.044	[−0.12, 0.06]	−0.696	0.487
Forcing another adult to engage in a sexual act with me × context	0.021	0.079	[−0.14, 0.18]	0.267	0.789
Submission PSC					
Being pressured into engaging in sex	−0.018	0.066	[−0.15, 0.11]	0.269	0.788
Being forced to do something sexually	−0.020	0.071	[−0.17, 0.11]	−0.374	0.709
Being sexually victimized	0.084	0.104	[−0.14, 0.27]	0.664	0.507
Being whipped or spanked	−0.020	0.048	[−0.11, 0.08]	−0.411	0.682
Being pressured into engaging in sex × context	0.059	0.061	[−0.06, 0.18]	0.973	0.331
Being forced to do something sexually × context	−0.029	0.065	[−0.16, 0.10]	−0.442	0.659
Being sexually victimized × context	0.019	0.096	[−0.17, 0.21]	0.204	0.838
Being whipped or spanked × context	0.021	0.044	[−0.07, 0.11]	0.485	0.628
Dominance NSC					
Forcing someone to do something sexually	−0.038	0.060	[−0.16, 0.08]	−0.637	0.525
Whipping or spanking someone	0.180	0.084	[0.02, 0.34]	2.146	0.032
Forcing another adult to engage in a sexual act with me	−0.020	0.062	[−0.14, 0.10]	−0.325	0.745
Forcing someone to do something sexually × context	−0.037	0.055	[−0.15, 0.07]	−0.675	0.500
Whipping or spanking someone × context	0.160	0.077	[0.01, 0.31]	2.078	0.038
Forcing another adult to engage in a sexual act with me × context	0.070	0.057	[−0.04, 0.18]	1.236	0.217
Submission NSC					
Being pressured into engaging in sex	−0.012	0.067	[−0.14, 0.12]	−0.181	0.857
Being forced to do something sexually	0.020	0.071	[−0.12, 0.16]	0.288	0.773
Being sexually victimized	0.084	0.061	[−0.04, 0.20]	1.382	0.168
Being whipped or spanked	−0.087	0.075	[−0.23, 0.06]	−1.161	0.246
Being pressured into engaging in sex × context	−0.101	0.061	[−0.22, 0.02]	−1.670	0.096
Being forced to do something sexually × context	0.120	0.065	[−0.01, 0.25]	1.844	0.066
Being sexually victimized × context	−0.030	0.056	[−0.14, 0.08]	−0.542	0.588
Being whipped or spanked × context	−0.123	0.069	[−0.26, 0.01]	−1.783	0.075

Note. PSC = positive sexual cognition; NSC = negative sexual cognition; *CI* = confidence interval.

## Data Availability

The dataset generated by the survey research is available at https://figshare.com/s/0d75980175760ac85a6d.
